# Unique Subclavian Vascular Ring Anomaly: Insights from CT Angiography

**DOI:** 10.3390/life15010077

**Published:** 2025-01-10

**Authors:** Radu Octavian Baz, Mihaly Enyedi, Cristian Scheau, Andreea Cristiana Didilescu, Radu Andrei Baz, Cosmin Niscoveanu

**Affiliations:** 1 Clinical Laboratory of Radiology and Medical Imaging, “Sf. Apostol Andrei” County Emergency Hospital, 900591 Constanta, Romania; andreibaz@yahoo.com (R.O.B.); raduandreibaz@yahoo.com (R.A.B.); cosmin_niscoveanu@yahoo.com (C.N.); 2 Department of Radiology and Medical Imaging, Faculty of Medicine, “Ovidius” University, 900527 Constanta, Romania; 3 Department of Radiology, “Victor Babes” Center for Diagnosis and Treatment, 030303 Bucharest, Romania; 4 Department of Anatomy, The “Carol Davila” University of Medicine and Pharmacy, 050474 Bucharest, Romania; 5 Department of Radiology and Medical Imaging, “Foisor” Clinical Hospital of Orthopaedics, Traumatology and Osteoarticular TB, 021382 Bucharest, Romania; 6 Department of Physiology, The “Carol Davila” University of Medicine and Pharmacy, 050474 Bucharest, Romania; 7 Department of Embryology and Microbiology, Faculty of Dentistry, The “Carol Davila” University of Medicine and Pharmacy, 8 Eroii Sanitari Boulevard, 050474 Bucharest, Romania; andreea.didilescu@umfcd.ro

**Keywords:** aortic arch, vascular ring, aberrant right subclavian artery, embryology, computed tomography, angiography, morphology, medical imaging

## Abstract

Aortic arch anomalies represent a range of congenital vascular malformations resulting from disruptions in the typical embryological development of the aortic arch and its branches. These anomalies, which vary widely in their presentation, can lead to significant clinical symptoms depending on their structure and position. We report the case of a 75-year-old male with intermittent hypertension, palpitations, and episodic warmth in the upper body. Computed tomography (CT) angiography revealed an atypical aortic arch anatomy with a unique right subclavian artery anomaly. The aortic arch displayed a typical orientation but included an additional arterial branch arising from the medial wall of the descending aorta. This aberrant branch with a tortuous aspect coursed posteriorly around the esophagus and merged with the subclavian branch of the brachiocephalic trunk, forming a vascular ring. A possible embryological hypothesis requires the persistence of both the distal segment of the right dorsal aorta and the right seventh intersegmental artery, as well as the right fourth aortic arch; however, the imaging aspect of our patient is not that of a classic double aortic arch. This case emphasizes the importance of advanced imaging techniques, such as CT angiography, in identifying and managing rare vascular anomalies that may influence patient care and clinical outcomes.

## 1. Introduction

Aortic arch anomalies encompass a diverse group of congenital vascular malformations that can significantly impact clinical outcomes. These anomalies often arise from deviations in the normal embryological development of the aortic arch system, leading to atypical branching patterns that may cause symptoms ranging from mild to severe depending on the nature and extent of the vascular irregularity [1,2].

The embryological development of the aortic arch involves a complex process of formation, regression, and remodeling of the six paired pharyngeal arch arteries [3]. Normally, the right fourth pharyngeal arch artery contributes to the right subclavian artery, and the left fourth pharyngeal arch forms a segment of the aortic arch [4]. Any deviation from this process can result in abnormal vascular structures, such as an aberrant subclavian artery [5].

## 2. Case Presentation

We hereby report a case of a 75-year-old male patient complaining of intermittent arterial hypertension, palpitations, and occasional warmth in the upper half of the body, for which he was recommended to undergo a cervical CT angiography.

**Figure 1:**
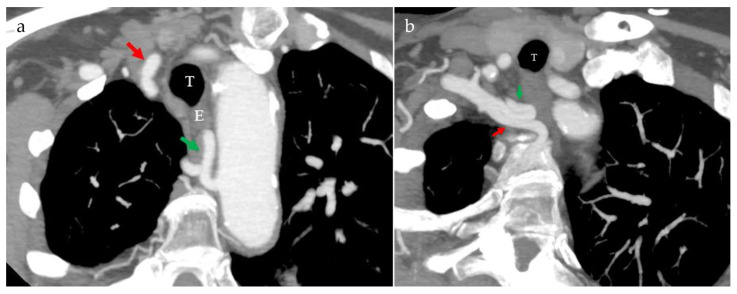
Maximum intensity projection images (**a**,**b**) of the CT scan show two arterial branches, the first one arising from the brachiocephalic trunk (red arrows) and the second one originating from the medial wall of the aortic arch (green arrows). Both vessels have a tortuous course, coiling around the esophagus and trachea, eventually merging to form the true right subclavian artery, which then follows its anatomical path. E = esophagus T = trachea.

**Figure 2:**
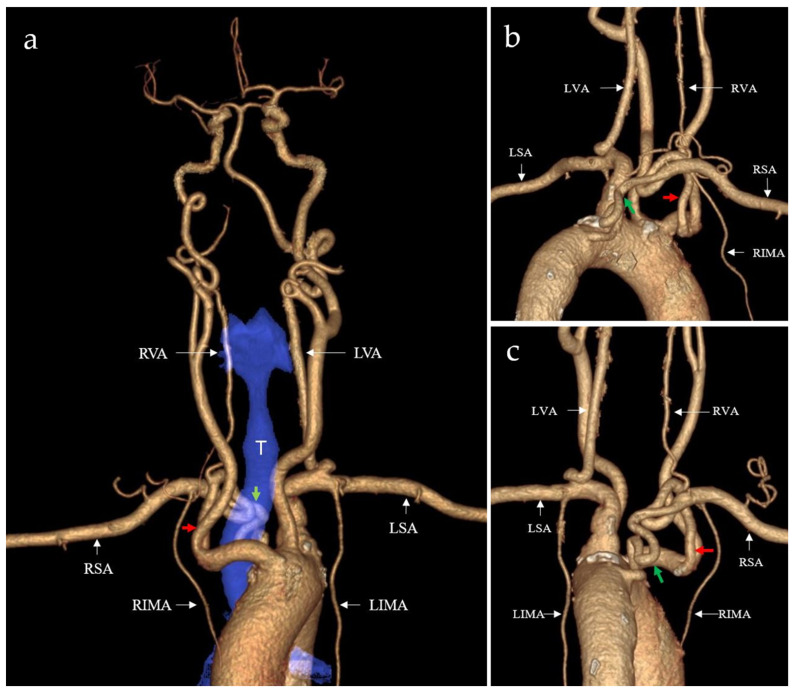
Virtual rendering anterior (**a**) and oblique (**b**,**c**) views revealed a normally oriented aortic arch from which the three main supra-aortic vessels emerged, and then a fourth branch similar to the lusoria artery had its origin. The origin and tortuous course of the aberrant branch can be identified (green arrows), along with its fusion with the true subclavian artery (red arrow). There was no significant compression effect of the two right subclavian branches over the esophagus or trachea (T, blue overlay). Also, there were no notable atherosclerotic changes to the aortic arch or any of the supra-aortic trunks. Origin of the internal mammary artery in the true RSA and of the vertebral artery from the brachiocephalic branch of the RSA. LSA = left subclavian artery;; RSA = right subclavian artery; RIMA = right internal mammary artery; LIMA = left internal mammary artery; RVA = right vertebral artery; LVA = left vertebral artery; T = trachea.

The examination revealed a normally oriented aortic arch from which, in order, the brachiocephalic trunk, the left common carotid artery, the left subclavian artery, and, finally, an arterial branch similar to the lusoria artery originated (Figure 1). This last branch emerged from the medial wall of the descending part of the aortic arch, had a tortuous course around and behind the esophagus, and joined the subclavian branch of the brachiocephalic trunk to form the true subclavian artery (Figure 2, Video S1).

The peculiarity of the case lies in the double origin of the true right subclavian artery and the vascular ring formed by the two branches. To the best of our knowledge, this vascular anomaly has not previously been observed in the literature.

## 3. Discussion

The vascular anomaly encountered in our patient may be understood by reviewing the embryological steps of aortic arch development.

In the intrauterine period, two ventral aortas are formed from the aortic sac. They follow the cranio-caudal folding of the embryo, thus being continued by the two dorsal aortas. At the end of the second month, these two vessels fuse on the midline, inferior to the seventh intersegmental artery, while the right dorsal aorta between the seventh intersegmental artery and the fusion of the dorsal arteries becomes atrophic and disappears [6]. Furthermore, arterial structures called arches are created in the branchial arches’ mesenchyme, which create anastomoses between dorsal and ventral aortas [7]. Arches I and II create the maxillary and stapedian arteries, respectively. Arch V becomes atrophic, without any mature derivatives. Arch III together with the dorsal aorta above will create the internal carotid artery, while the ventral aorta between arches III and IV will become the common carotid artery. The external carotid artery will emerge from the anterior aspect of arch III. The left ventral aorta becomes the ascending aorta and is continued by the left arch IV, which becomes the aortic arch, followed by the left dorsal aorta. The right arch IV together with the right dorsal aorta and the seventh intersegmental artery becomes the right subclavian artery. The right ventral aorta inferior to arch IV becomes the innominate artery, while the left subclavian artery is formed only by the seventh intersegmental artery [8,9,10,11,12].

Vascular rings are a group of congenital anomalies of the aortic arch characterized by the abnormal development of the arch and its branches, leading to the formation of structures that encircle the midline mediastinal structures—the esophagus and/or trachea [13]. There are several ways of grouping the aortic arch anomalies, most commonly into complete (double aortic arch and right aortic arch with the left ligamentum arteriosum) and incomplete (aberrant subclavian artery and Kommerell’s diverticulum) vascular rings. Right aortic arch anomalies were also described, as well as left aortic arches with an aberrant right subclavian artery (ARSA) and an aberrant left pulmonary artery [14,15]. Several studies attempted to systematically classify these anomalies [16,17,18,19]. However, we found that our reported anomaly does not fit any of the previously reported classifications.

**Figure 3:**
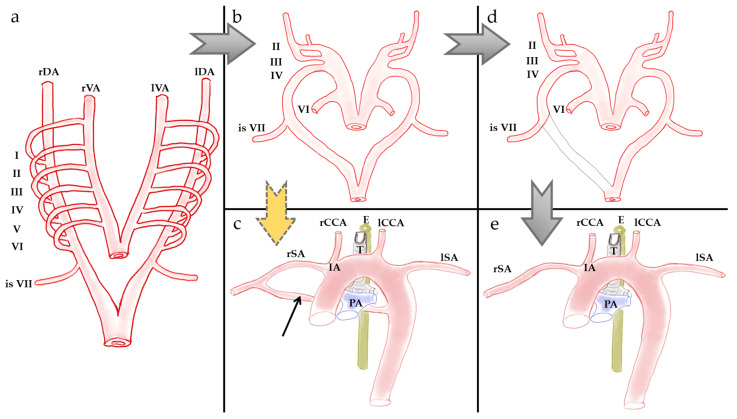
Schematic of the development and evolution of the aortic arches in normal embryology (black arrows) and in our case (yellow arrow). (**a**) Aortic arches in the 4th week of development. I—1st aortic arch; II—2nd aortic arch; III—3rd aortic arch; IV—4th aortic arch; V—5th aortic arch; VI—6th aortic arch; VII—7th right intersegmental artery. (**b**,**d**,**e**) Normal development of the aortic arches, 5th week of development. II, III, VI—corresponding arterial arches. VII—7th right intersegmental artery. rDA—right dorsal aorta; lDA = left dorsal aorta; rVA = right ventral aorta; lVA = left ventral aorta; rSA—right subclavian artery, derived from the right fourth aortic arch, the right dorsa aorta, and the 7th intersegmental artery; rCCA—right common carotid artery, derived from the right ascending aorta between arches IV and III; E—esophagus; T—trachea; lCCA—left common carotid artery, derived from the left ascending aorta between arches IV and III; lSA—left subclavian artery, derived from the left 7th intersegmental artery; IA—innominate artery, derived from the right ascending aorta between arches VI and IV; PA—pulmonary artery. (**c**) Development variant with the persistent right dorsal aorta inferior to VII, forming together with the aortic arch and the rSA an arterial ring around the trachea and esophagus (black thin arrow).

In our patient’s case, the right subclavian artery is formed by the convergence of two arterial branches, the first coming from the brachiocephalic trunk and the second emerging from the distal portion of the aortic arch. The union of these two vessels forms a wide vascular ring. The aberrant origin of the right subclavian artery from the distal portion of the aortic arch is based on the abnormal regression of the right dorsal aorta. Instead of the distal segment involution, this segment persists, together with the partial regression/persistence of the proximal two-thirds of the right dorsal aorta, along with the proximal portion of the right fourth aortic arch [20]. Thus, the right subclavian artery will arise from two sources: the innominate artery and the distal portion of the aortic arch. On the other hand, in the case of the formation of a vascular ring similar to a double aortic arch, the persistence of the entire right fourth aortic arch is necessary (Figure 3).

To summarize, a possible embryological hypothesis for this variant would require the persistence of both the distal segment of the right dorsal aorta and the right seventh intersegmental artery (to explain the vascular branch emerging from the distal third of the aortic arch, similar to the arteria lusoria), as well as the right fourth aortic arch (which would contribute to the formation of the vascular branch detached from the brachiocephalic trunk, with an elongated and curved course to the retroesophageal level, where it joins the one coming from the aortic arch). However, the imaging aspect of our patient is not that of a classic double aortic arch.

During embryonic development, blood flow patterns can influence vascular development [21,22]. The onset of blood flow completely changes the environment in which the cardiovascular system is formed, providing physiological feedback, so that the developing vascular network is in a continuous state of adaptation [21,22,23]. Although this aspect has not been studied extensively, studies in the literature [24,25] suggest that blood fluid hemodynamics and flow disturbances are possible mechanisms with an impact on vascular development, potentially influencing events such as the regression and formation of aortic arches.

The correct and early identification of specific vascular anomalies or anatomic variants may play a major role in patient management considering the potential impact on the diagnosis and treatment of subsequent pathologies [26,27,28,29]. With the advent of advanced imaging technologies, enhanced visualization applications, and 3D printing capabilities, complex anatomic variants are easier to identify and explore with better capabilities of preoperative simulation and training in selected cases [30,31]. With the advent of emerging materials and the increasing capability of additive-manufacturing technologies, 3D printing may have the potential to also serve as a therapeutic option for manufacturing vascular or supportive structures with biological properties where required [32,33,34,35,36,37,38].

The vascular ring abnormalities of the aortic arch can occur in isolation but may al-so be associated with cardiac and/or chromosomal defects, particularly conotruncal anomalies, trisomy 21 (Down syndrome) or trisomy 18 (Edwards syndrome) [39,40]. A nonrecurrent inferior laryngeal nerve is a very important associated anomaly, especially with an aberrant right subclavian artery (ARSA), when the vagus nerve directly innervates the larynx, instead of swinging down below the arch [41,42]. An aberrant right thoracic duct draining into the venous angle can accompany some vascular rings [42].

The majority of patients with an ARSA are asymptomatic, as this anomaly is usually discovered accidentally and requires no further treatment. Symptoms such as respiratory distress and dysphagia usually occur in infancy under significant compression of the trachea or esophagus by the vascular rings [43,44]. In adults, the constriction of the esophagus may progress due to the loss of vascular compliance, resulting in the delayed onset of symptoms in patients with vascular rings [45]. Causes of arterial stiffening include atherosclerotic plaque deposition, systemic hypertension, decreased connective tissue elasticity, and decreased smooth muscle relaxation. Albeit asymptomatic, the identification of a vascular ring should alert the radiologist and surgeon due to the associated anomalies [46]. A nonrecurrent inferior laryngeal nerve can be injured in carotid artery or thyroid procedures if this anomalous course is not suspected. An aberrant course of the thoracic duct associated with some vascular rings can lead to unwanted injuries of the duct in thoracic surgery. In addition, in cases of an ARSA, clamping the aorta proximal to the left subclavian artery during surgery will occlude both vertebral arteries, which will lead to brainstem infarction. In trauma, an aortic dissection may extend into the aberrant retroesophageal artery, which may perforate into the esophagus and lead to exsanguination [47,48,49,50]. In our case, there was no evidence of compression of the esophagus by the vascular ring and the aberrant branch was free of any visible lesions. This might be attributed to the particular features of this unique anatomic variant, including its tortuous course and thin caliber, making it less likely to impact adjacent structures compared to a larger, thicker, and straighter vascular lumen, as commonly observed in right aortic arch anomalies.

Barium esophagrams, ultrasonography (US), CT, magnetic resonance imaging (MRI), and catheter angiography are the imaging techniques used to identify and diagnose aortic arch variants and anomalies [51,52,53]. Classical modalities such as barium esophagrams and catheter angiography are not frequently used as first-line diagnostic tools because they are difficult to use in pediatric patients and provide only two-dimensional information [54]. So, at present, echocardiography, MRI, and CT angiography remain the main techniques used to detect and evaluate vascular rings [15,55].

Pediatric anomalies, anatomic variants, and congenital disorders may be associated with increased mortality and often require complex surgery and enhanced recovery protocols [56,57,58]. In the setting of vascular rings, transthoracic echocardiography is often the initial imaging modality used. US poses minimal risk to the patient, as it is noninvasive, does not require use of intravenous contrast medium, does not expose the patient to radiation, and is readily available at the bedside [59,60]. Uncomplicated arch anomalies in infants and toddlers may be investigated with echocardiography alone. However, cross-sectional imaging with CT angiography is the modality of choice for diagnosing vascular rings due to its rapidity, high spatial resolution, and the availability of multiplanar image reformatting and volume rendering techniques [52]. Reformation in multiple planes or three-dimensional imaging can provide detailed anatomical information, including relative positional relationships, which is advantageous, especially when assessing tracheal and esophageal compression. It is usually performed without gating, which reduces the exposure together with advanced radiation dose reduction technologies [52,55]. MRI is an alternative cross-sectional imaging modality that allows for the precise assessment of aortic arch anomalies without ionizing radiation [55]. Contrast-enhanced MR angiography and non-contrast-enhanced black blood and bright blood sequences can be performed to evaluate anatomical relationships between the aortic arch, esophagus, and the airways. [61]. Similarly to CT, 3D volumetric acquisitions provide multi-planar reformatting for detailed assessment of tracheal compression [62]. However, the most significant drawback of MRI is the long acquisition times. To overcome this limitation, several techniques, such as compressed sensing, 3D ultra-short echo time, and 4D flow acquisition are being used [63]. In our case, a CT angiography was the method of choice due to its availability, speed of acquisition, and high-quality expected outcome.

The treatment of vascular rings depends on the severity of the symptoms. Patients with only mild esophageal or tracheal compression with no signs of respiratory distress or dysphagia are managed conservatively, as in our case. More severe symptoms may require surgical intervention with a vascular ring division to relieve the compressive effects [9,64]. Recent improvements in the surgical therapeutic field, such as local or remote robotic surgery which may be combined with artificial intelligence, could play a role in the future of this pathology, ensuring shorter hospital stays, fewer complications, and better outcomes [65,66,67,68].

The identification of rare anomalies of the aortic arch, such as the subclavian vascular ring described in this case, has critical clinical implications beyond immediate patient symptom presentation. As very well highlighted by Dumfarth et al. [69], vascular anomalies can represent potential biomarkers for the underlying aortic pathology or a predisposition to thoracic aortic disease. Although our patient presented with palpitations and intermittent hypertension, the identification of this anomaly could warrant long-term cardiovascular surveillance. Risk stratification and possible earlier intervention in the case of aneurysm development or structural vessel changes are made possible by the proactive identification of this variant [70]. Moreover, Takayama emphasized the necessity for recognizing congenital anomalies as possible indicators of a broader cardiovascular pathology [71]. A thorough understanding of these anomalies can represent a basis for decision-making and support the guidance and management of follow-ups and surgical planning [72].

This case underlines the importance of advanced imaging techniques such as CT angiography in the detection of anatomic variants with potential broader implications. Future research should assess the embryological basis of these anomalies and evaluate their potential to serve as biomarkers for cardiovascular pathology. Genetic studies on the pharyngeal arch artery development could provide additional information regarding the pathogenesis, implications, and new and better therapeutic approaches of these conditions [73]. Future imaging studies on a larger scale that include asymptomatic as well as symptomatic patients could properly assess the prevalence and association of these anomalies with long-term cardiovascular risk [74]. The development of adequate guidelines for the surveillance and approach of these incidental cases could prove to be very valuable, particularly in subgroups of patients already carrying other risk factors [75].

## 4. Conclusions

This case report highlights the clinical significance of aortic arch anomalies and their potential to cause symptomatic vascular rings. While our reported case did not exhibit any specific symptoms related to the anatomic variant, the unique presentation of an aberrant right subclavian artery with a dual origin and a tortuous, retroesophageal course of one of its branches accentuates the complexity of these anomalies and their potential impact on patient health.

CT angiography, as an advanced imaging technique, is of utmost importance in diagnosing complex vascular anomalies. The accurate identification of these anomalies is crucial for effective management and treatment, which may involve surgical or endovascular interventions to alleviate symptoms and prevent complications. Future studies are needed to further understand the genetic and molecular mechanisms underlying these developmental anomalies and to improve diagnostic and therapeutic approaches. Increased awareness and recognition of such vascular anomalies can lead to better clinical outcomes for affected patients.

## Data Availability

The data presented in this study are available upon reasonable request from the corresponding authors.

## Supplementary Materials

The following supporting information can be downloaded at: https://www.mdpi.com/article/10.3390/life15010077/s1, **Video S1**: Video rendering technique of the vascular anomaly; trachea is featured in a blue overlay.
